# Who should decide for local health services? A mixed methods study of preferences for decision-making in the decentralized Philippine health system

**DOI:** 10.1186/s12913-020-05174-w

**Published:** 2020-04-15

**Authors:** Harvy Joy Liwanag, Kaspar Wyss

**Affiliations:** 1 Swiss Tropical and Public Health Institute, Basel, Switzerland; 2 Balik Scientist Program, Department of Science and Technology Philippine Council for Health Research and Development (DOST PCHRD), Metro Manila, Philippines; 3 Ateneo de Manila University School of Medicine and Public Health, Metro Manila, Philippines; 4 University of Basel, Basel, Switzerland

**Keywords:** Decentralization, Decision making, Health policy, Mixed methods, Philippines, Public health administration

## Abstract

**Background:**

The Philippines decentralized government health services through devolution to local governments in 1992. Over the years, opinions varied on the impact of devolved governance to decision-making for local health services. The objective of this study was to analyze decision-makers’ perspectives on who should be making decisions for local health services and on their preferred structure of health service governance should they be able to change the situation.

**Methods:**

We employed a mixed methods approach that included an online survey in one region and in-depth interviews with purposively-selected decision-makers in the Philippine health system. Study participants were asked about their perspectives on decision-making in the functions of planning, health financing, resource management, human resources for health, health service delivery, and data management and monitoring. Analysis of survey results through visualization of data on charts was complemented by the themes that emerged from the qualitative analysis of in-depth interviews based on the Framework Method.

**Results:**

We received 24 online survey responses and interviewed 27 other decision-makers. Survey respondents expressed a preference to shift decision-making away from the local politician in favor of the local health officer in five functions. Most survey participants also preferred re-centralization. Analysis of the interviews suggested that the preferences expressed were likely driven by an expectation that re-centralization would provide a solution to the perceived politicization in decision-making and the reliance of local governments on central support.

**Conclusions:**

Rather than re-centralize the health system, one policy option for consideration for the Philippines would be to maintain devolution but with a revitalized role for the central level to maintain oversight over local governments and regulate their decision-making for the functions. Decentralization, whether in the Philippines or elsewhere, must not only transfer decision-making responsibility to local levels but also ensure that those granted with the decision space could perform decision-making with adequate capacities and could grasp the importance of health services.

## Background

Decentralization of health services, particularly in the form of devolution of authority to local governments [1], has been implemented in many countries motivated by the expectation that it will empower local decision-makers to oversee and steer their own health services [2]. Yet beyond a few studies of selected disease-specific programs where decentralization resulted in better health outcomes [3, 4], decentralization’s effectiveness in improving outcomes related to the broader dimension of health system performance is uncertain based on the limited number of reviews that examined the global evidence [5–7]. On the other hand, the fact that local decision-makers are granted the “decision space” [8, 9], which provides an idea of the extent of choices available to them, and are able to make decisions for the functions of local health services because of decentralization may already be a desired outcome in itself. Therefore, when analyzing the effectiveness of decentralization, it is important to take into account the perspectives of local decision-makers themselves—how they perceive and utilize their decision space in relation to other system actors, as well as how they might want to modify the decentralized structure of governance of health services given their experience in navigating the system. This study was driven by such a desire to examine the perspectives of decision-makers in the Philippines where government health services were decentralized through devolution to local governments since 1992 [10, 11]. These local governments enjoy a level of autonomy from the central government and are comprised of provinces and, within these, the municipalities and cities.

A number of studies, drawing from the decision space framework, have assessed the extent of autonomy enjoyed by local decision-makers in countries that have decentralized or devolved their health systems [12–17]. Most have attempted to come up with an overall or summative assessment of local decision space yet, in practice, decision-making for health services often involve multiple actors, each of whom may be wielding varying degrees of influence into the decision-making process. Thus, we have previously deconstructed local decision space qualitatively between politicians and health managers (i.e. political and technical decision-makers) in the Philippines and concluded that the latter’s decision space for various functions was narrower than the former’s [18]. It is therefore policy-relevant to examine whether or not decision-makers in the Philippines would prefer to shift the extent of decision space available between types of decision-makers in order to improve decision-making for local health services, defined as the range of healthcare services (i.e. promotive, preventive, curative, and rehabilitative) being provided in local health facilities.

Despite more than two decades of efforts to evolve towards more decentralized operations in the Philippines, there has been no clear evidence in the peer-reviewed literature on whether or not and to what degree devolution has influenced health system performance. Nevertheless, a lot has changed in the Philippines: the population grew to 105 million in 2017 from only 62 million in 1990; life expectancy at birth increased to 69.1 in 2016 compared to 65.3 in 1990; cardiovascular diseases became the leading cause of mortality by 2014 while infectious diseases like pneumonia ranked fourth; infant mortality rate in 2016 was down to 21.5 per 1000 compared to 40.8 in 1990 [19]. Yet there still remains a divergence of positions in the Philippines between those, on one side, who desire to expand decentralization even further by granting local governments greater control (i.e. wider decision space) over financial resources to better support local health facilities [20] and those, on the other side, who want the central government to re-take ownership and management of local health facilities by reversing devolution and re-centralizing the system [21, 22]. To our knowledge, no other study in the peer-reviewed literature has explored on which side of this divergence local decision-makers in the Philippines actually stand—i.e., whether they prefer to maintain the *status quo,* or prefer to change the current structure of governance. As mentioned, the evidence for decentralization’s effectiveness is mixed, and so it remains to be seen which between a more decentralized or a more centralized structure of governance would improve health system performance. However, if decentralization is indeed about enabling localities to have a say on how decisions for health services are made, it is important to ensure that the views of local decision-makers themselves, who are most familiar with the issues surrounding health service decentralization at local levels, are considered.

Thus, the objective of this study was to analyze the perspectives of local decision-makers in one region in the Philippines on who should be making decisions for health service functions at local levels, as well as their preferences for the decentralized governance structure of health services. This study aimed to further analyze the preferences by triangulating these with the perspectives of decision-makers representing various levels of governance in the Philippine health system.

## Methods

### Mixed methods approach

This study employed a mixed methods approach. We were guided by the Framework Method [23] of qualitative health research, which involved the steps of transcription, familiarization with the interviews, coding, developing and applying an analytical framework, and charting and interpreting the data. We developed an interview guide that allowed in-depth exploration of how decision-makers make decisions for selected health service functions (described later), including their flexibility in decision-making for each function to estimate their decision space, and the various local actors whom they perceived to be involved in decision-making. This interview guide was previously published [18].

For the quantitative approach, an online survey explored decision-makers’ understanding of devolution, their opinion on its benefits to the delivery of health services, as well as their perceived challenges in its implementation. There was a two-step approach in the survey to inquire about decision-making for each function, such that a question first asked the respondents to identify who among local decision-makers they perceived to have the widest decision space (i.e. exerts the most influence over decision-making for that particular function). This was followed by a question that asked them to identify whom they would rather prefer to be influencing the decisions instead. A copy of the survey questionnaire is provided as an online supplement (see Additional file 1).

The survey questionnaire then asked respondents for their preferred governance structure of the health system—i.e., whether they would prefer to maintain the current devolved system, or would prefer to modify the governance structure by adopting options for re-centralization. Responses were collected through Google Forms (https://www.google.com/forms/about/).

### Decision-makers

We performed the in-depth interviews face-to-face with up to 27 decision-makers in 2017 who were purposively-selected to represent different levels of decision-making, institutional affiliations, and geographical settings in the Philippine health system. The profiles of these 27 decision-makers [18] and a selection of quotes from our interviews with them [24] were published elsewhere. It was at the 27th interview when saturation [25] was judged to have been achieved, or the point when new information was no longer emerging. Saturation likewise ensured that the themes identified in the analysis were recurring rather than isolated themes. The interviews lasted 1 h on average and were transcribed in Microsoft Word 2016 in preparation for qualitative analysis.

We then performed the online survey for local decision-makers in the region of Northern Luzon in the Philippines. The region was selected out of convenience because of the first author’s (HJL) contacts with key officials of the Department (i.e. Ministry) of Health (DOH) regional office who supported this study and provided access to records. Northern Luzon is composed of the four provinces of Ilocos Norte, Ilocos Sur, La Union, and Pangasinan, together with the 116 municipalities and nine cities within these provinces, and has a combined population of five million [26]. We defined a “decision-maker” for this study as someone who is in a position, whether elected (i.e. political) or appointed/career (i.e. technical), who participates in performing six selected health service functions. Following this definition, we targeted the following groups of local decision-makers and obtained a list of their email addresses from the DOH regional office:

- *Technical:* DOH regional office staff deployed to the various local governments in the region to provide technical assistance (e.g. “Development Management Officers” or DMOs, “Public Health Associates” or PHAs, “Nurse Deployment Program” or NDP nurses, and medical doctors under the “Doctors to the Barrios” (DTTB) program;
- *Political:* Elected officials who head the local governments (e.g. provincial governors, municipal and city mayors, and other local politicians); and
- *Technical:* Local health managers employed by the local governments to supervise and manage health services (e.g. provincial, municipal, and city health officers who are also medical doctors).

**Fig. 1:**
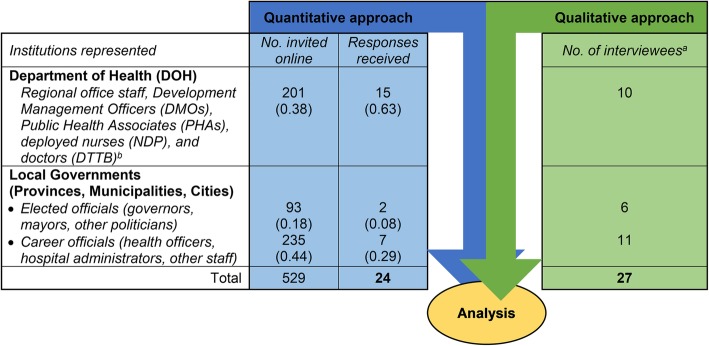
Number of respondents for the mixed methods study. *Legend: a – Profiles of interviewees are available in Liwanag and Wyss* [18]*. b – DMOs are DOH staff who liaise with the local governments to advocate for the attainment of health objectives and provide technical assistance* [27]*; PHAs are DOH staff deployed to local governments to assist primarily in data collection* [28]*; NDP nurses* [29] *and DTTB medical doctors* [30] *are DOH-hired staff who are deployed to local health facilities to provide services and augment the local governments’ lack of human resources*

The online survey questionnaire was sent to the 682 email addresses in the list. There were 153 emails that bounced back or failed to deliver, resulting in a total of 529 valid emails for follow-up. Email reminders were sent at least twice during a three-month period in 2018. In the end, we received 24 responses, or a response rate of 4.5%. Figure 1 provides a summary of the number of respondents for the quantitative approach (online survey) and the qualitative approach (in-depth interviews) (Fig. 1).

### Health service functions

The analytical framework was based on six selected health service functions, which we defined as broad categories of tasks that involve decision-making for health services. These functions were drawn from the interviews following a deductive approach and grouped into the following categories:

- *Planning* – development by the local health board or committee of plans for health services, including deciding which needs to prioritize and address;
- *Health financing* – determining the budget allocation for local health services, including the sources of funds and how to spend the budget;
- *Resource management* – making decisions on the construction of new (or renovation of existing) local health facilities, including the procurement and maintenance of equipment and supplies (e.g. medicines) to make such facilities functional (Note: in the Philippines, these local health facilities are owned and managed by local governments, such as the provincial and district hospitals which are under the provinces and the primary care facilities called “Rural Health Units” or RHUs which are under the municipalities; cities may own both hospitals and RHUs);
- *Human resources for health* – hiring and firing of local health staff, including making decisions related to supporting their training and providing the range of benefits that they are entitled to;
- *Health service delivery* – deciding what range of health programs (e.g. maternal and child health program, reproductive health program) and services (e.g. immunization, TB-DOTS) will be made available in local health facilities;
- *Data management and monitoring* – deciding which indicators to collect and how to use the data for monitoring the performance of local health services.

Quantitative analysis involved visualizing the trends in the survey responses through the use of radar charts in Microsoft Excel 2016. To triangulate survey findings, we brought in the themes from the qualitative analysis of the in-depth interviews, which was performed in MAXQDA Standard 12 (VERBI GmbH Berlin 2018). We reviewed the interview transcripts to identify illustrative quotes that may explain the results of the online survey.

## Results

### Study participants

There were 17 male and 10 female participants in the in-depth interviews with an average of 24 years of service in the government sector in various locations across the Philippines [18]. There were 22 medical doctors, three lawyers, and two non-health professionals. Most were local government health officers (11/27) and DOH staff serving in various capacities (10/27), and the rest were politicians (6/27). As the goal of qualitative research is a deeper understanding of participants’ insights and their context, purposive selection that obtained a wide variation in the profiles of participants rather than statistical representation was the sampling approach [31].

**Fig. 2:**
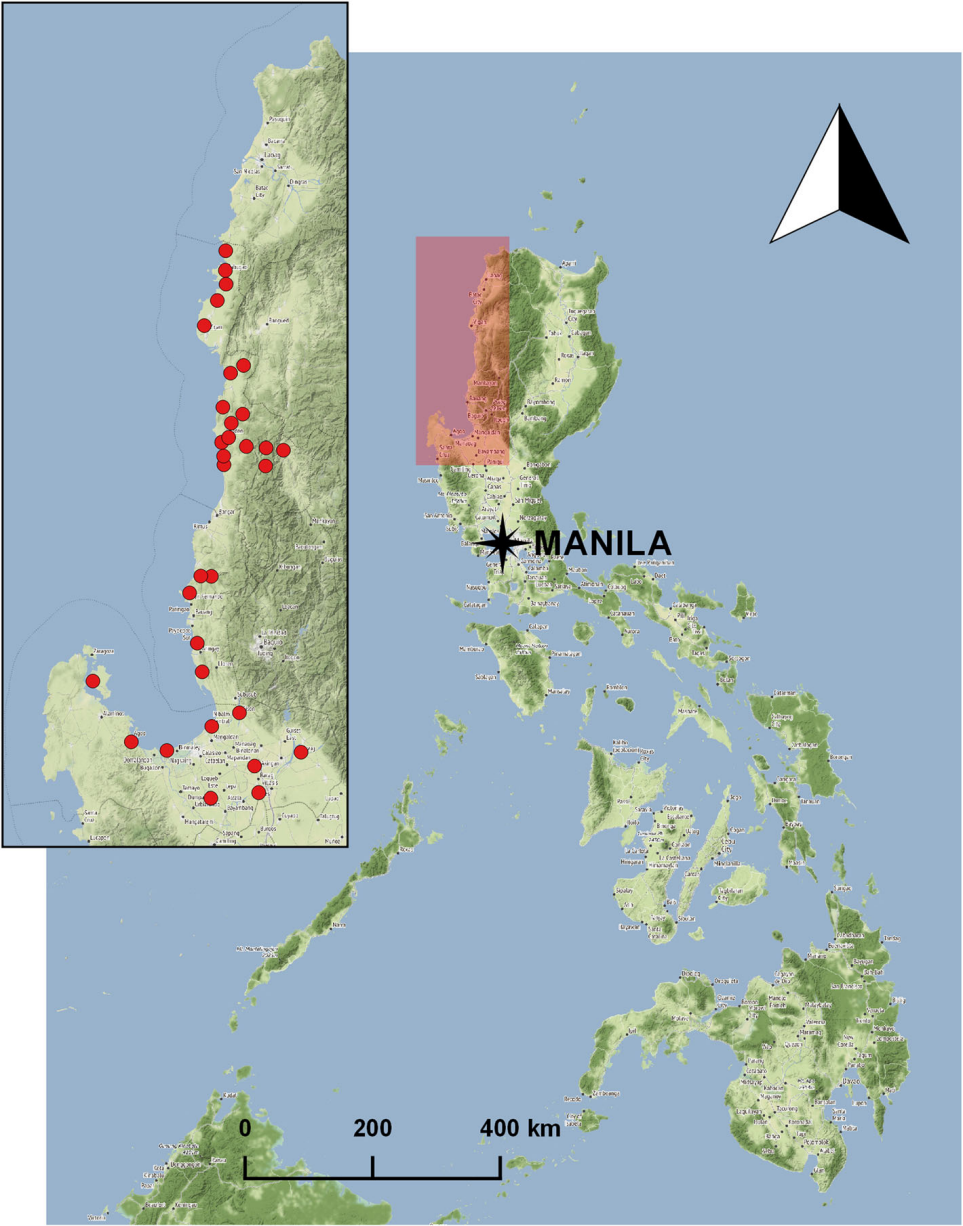
Work locations of the survey respondents in Northern Luzon, Philippines. *(Map tiles by Stamen Design, under CC BY 3.0. Data by OpenStreetMap, under ODbL)*

On the other hand, there were 14 female and 10 male online survey participants with an average of 14 years of serving in government. There were 12/24 nurses, 10/24 medical doctors, and 2/24 non-health professionals. Mapping their work locations suggested a good coverage of local settings in the region of Northern Luzon. Some survey respondents had the responsibility for multiple local governments, which explains why there were > 24 locations plotted on the map (Fig. 2).

Most survey respondents were DOH staff deployed to the local governments in the region (15/24), followed by local government health officers (7/24), while two were local politicians (Fig. 1). Consequently, in our discussion of results below, we would interpret survey results as a reflection of the perspectives mainly of DOH staff and local health officers and less of politicians. The anonymized dataset of survey responses is also available as an online supplement (see Additional file 2).

### Perspectives on devolution

When asked about the benefits of devolution to health services, more than half (13/24) of survey respondents answered *“empowerment of local governments to decide for themselves and address their own health needs.”* On the other hand, their responses to the challenges in implementing devolution were more varied, such that 6/24 answered *“decisions related to local health services have become politicized rather than evidence-based,”* another 6/24 answered *“local governments have continued to depend on assistance from the DOH,”* and 5/24 answered *“local health workers’ full range of compensation and benefits has not been provided consistently.”*

### Political vs. technical decision-maker

**Fig. 3:**
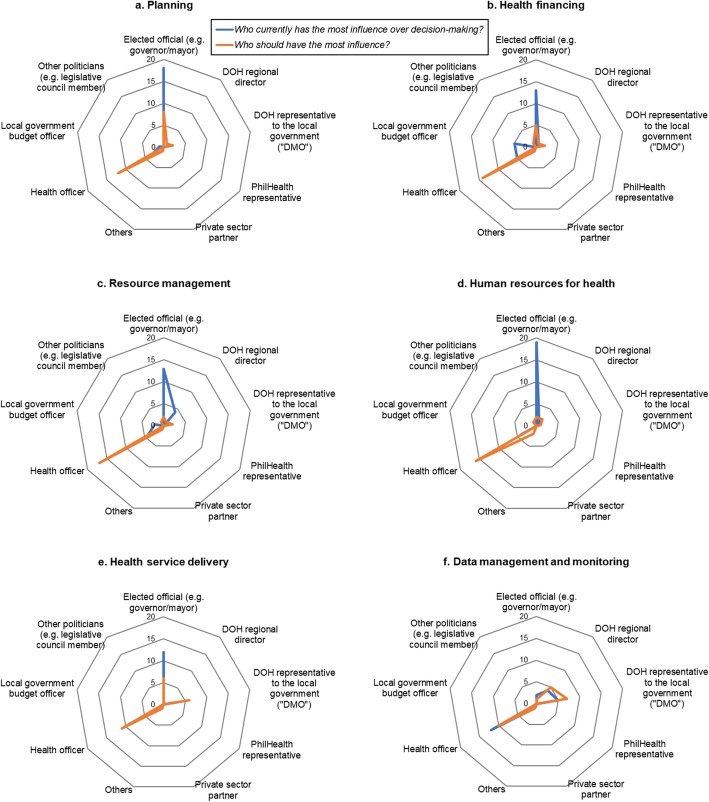
Decision-makers who were perceived to be influencing decision-making for the various functions of local health services

Visualizing the responses on radar charts for the six functions revealed that most survey participants perceived decision-making to be currently influenced by the local politician (i.e. the provincial governor or municipal/city mayor has the wide decision space), but would rather have the local health manager (i.e. provincial, municipal, or city health officer) to influence decision-making for the functions instead. This preference to shift the decision space away from the local politician in favor of the local health officer was consistent in five functions (Fig. 3). Illustrative quotes from the interviews were provided for each function below to shed some light on the potential reasons on why survey respondents expressed this preference.

### Planning

Survey respondents perceived the governor or mayor to have the most influence over decisions related to planning, yet most of them would prefer the health officer to influence the decisions for this function instead. Decision-making in planning relies on the participation of the governor or mayor to chair the meeting of the Local Health Board (LHB) where local health plans are discussed and approved. The interviews revealed difficulties in meeting regularly to plan together due to a lack of interest from the local politician, as illustrated by the following quote.

“*Our LHB is not really functional because we would set a meeting and the mayor is not around, although it is possible for somebody else to be the presider. But of course, what we want is for the mayor to be there because once he calls for it, the other members really participate.” – City health officer in a highly-urbanized city, 32 years in government.*

### Health financing

In health financing, survey respondents also identified the governor or mayor to be mostly influencing the decisions, yet would similarly prefer the health officer to influence the decisions for this function instead. The governor or mayor currently has the authority to approve the local government budget allocation for health services upon the recommendation of the health officer. However, the interviews indicated several instances when the health officer’s desired budget is not approved by the governor or mayor who may choose to prioritize other needs.

*“Before devolution, we were all health workers [under the DOH] and shared the same thinking and understood the mandate of a hospital, which is to serve the people. But now, the finance staff in my local government have a different outlook. They are more into saving money. I have to exert effort to convince them to fund our health programs. My decisions pass through the governor, and I need to convince him. Whereas, before devolution, as chief of the hospital I had the autonomy, I could already decide what to do with the money.” – Provincial health officer in a low-income province, 29 years in government.*

Nevertheless, limitations in local budget allocations could be addressed through financial augmentation from the national social health insurance program, which is managed by PhilHealth. An example of how this may be done was illustrated by the following:

*“In 2000, I had an annual budget of 300,000 pesos [USD ~6,000] for the entire municipality with a population of 9,000. The Sangguniang Bayan [town council] could not give us additional budget. What did I do? Instead of using the budget for medicines, I used it to enroll our constituents in PhilHealth at the cost of 120 pesos [USD 2.40] per family per year at that time. In return, PhilHealth would pay a capitation fund of 300 pesos [USD 6] per family. So around 180 pesos [USD 3.60] per family was returned to the local government as income. Instead of having 300,000 pesos, my budget increased to around 600,000 pesos, and we could use some of that to buy medicines too.” – Municipal health officer in a low-income municipality, 17 years in government.*

### Resource management

Decisions in resource management were also seen to be influenced the most by the governor or mayor according to survey respondents, but still preferred the health officer to influence the decisions instead. While the health officer is responsible for the maintenance and upgrade of local health facilities, including the equipment and supplies (e.g. medicines) needed therein, final decisions regarding these are made by the governor or mayor. The interviews revealed situations where the health officer would disagree with the governor or mayor and thus receive little local government support for resource management.

*“Every time I support a candidate during the elections, a different one wins as mayor. That is why all the improvements in our RHUs come from assistance from the central government through HFEP [Health Facilities Enhancement Program], and none from my local government. I once asked my mayor bluntly, ‘Mayor, why are you squeezing me? Why can’t we have a good relationship?’ It’s difficult. He’s always saying yes to me, but on the contrary, he is not giving support for facilities.” – Municipal health officer in a middle-income municipality, 28 years in government.*

This same health officer, however, also explained that challenges in infrastructure could be addressed with the support provided by the central government, as he illustrated further:

*“Through HFEP, I submit a proposal to the DOH. Low-income municipalities are prioritized in HFEP. I requested for a maternity and lying-in facility, which the DOH constructed for us. It’s not only the building, but we’re also given the equipment, like the delivery table. If my local government could not provide for the facilities, then I make proposals through HFEP.”*

### Human resources for health

In the management of human resources for health, the governor or mayor was also identified to be mostly influencing the decisions, yet the health officer was preferred by survey respondents. Currently, the governor or mayor has the final say on the appointment of local government health staff. The interviews revealed situations where the governor or mayor would decide on hiring based on political considerations rather than the qualifications of applicants.

*“I don’t always get the right person for the right job. When it comes to hiring, it’s still the governor who has the final say. Of course, there is patronage, whoever is close to the governor, whoever will be able to add to his votes, that’s the person who gets hired. I usually get consulted in hiring the professionals, for example, laboratory staff, or the positions with supervisory roles. But for the rank and file positions, it’s the governor who decides. These people however should also be capable. We should not just hire anybody who knows nothing.” – Provincial health officer in a low-income province, 29 years in government.*

### Health service delivery

Decisions in health service delivery were likewise perceived by survey respondents to be mostly influenced by the governor or mayor yet the health officer was the preferred decision-maker. While the health officer can propose health programs or services to be implemented in the locality, these will require the approval of the governor or mayor. The interviews revealed a tendency for the governor/mayor to support only those programs that result in political gains.

*“My mayor was not supportive of my anti-smoking initiative. In fact, he did not like it because he said that people will get mad, the stores will be mad. But I was able to convince him because I told him that he will get an award for this, that he will be recognized. So he supported it, not because he was thinking that disease can be prevented, but because he wants to get the award. But politicians are like that, they are for recognition, never mind the impact.” – Municipal health officer in a low-income municipality, 16 years in government.*

### Data management and monitoring

On the other hand, survey respondents perceived the health officer to be influencing the decisions in data management and monitoring and would also prefer the health officer to have a wide decision space for this function. The health officer is responsible for the timely and accurate collection of data in the locality, which are then pooled at provincial, regional, and central levels through coordination with the DOH offices at various levels. As illustrated below, the interviews did not indicate interference by politicians in data management and monitoring, which may explain the lack of divergence between current and preferred decision-maker for this function.

*“Our data are updated, partly-paper and partly-electronic. We submit our reports to the DOH through the public health associates. Because gathering the data is important, we make sure that every pregnant woman is monitored at the level of the Barangay (village). We also know the indicators where we are lagging behind.” – Municipal health officer in a low-income municipality, 17 years in government.*

### Preferences for governance structure

**Fig. 4:**
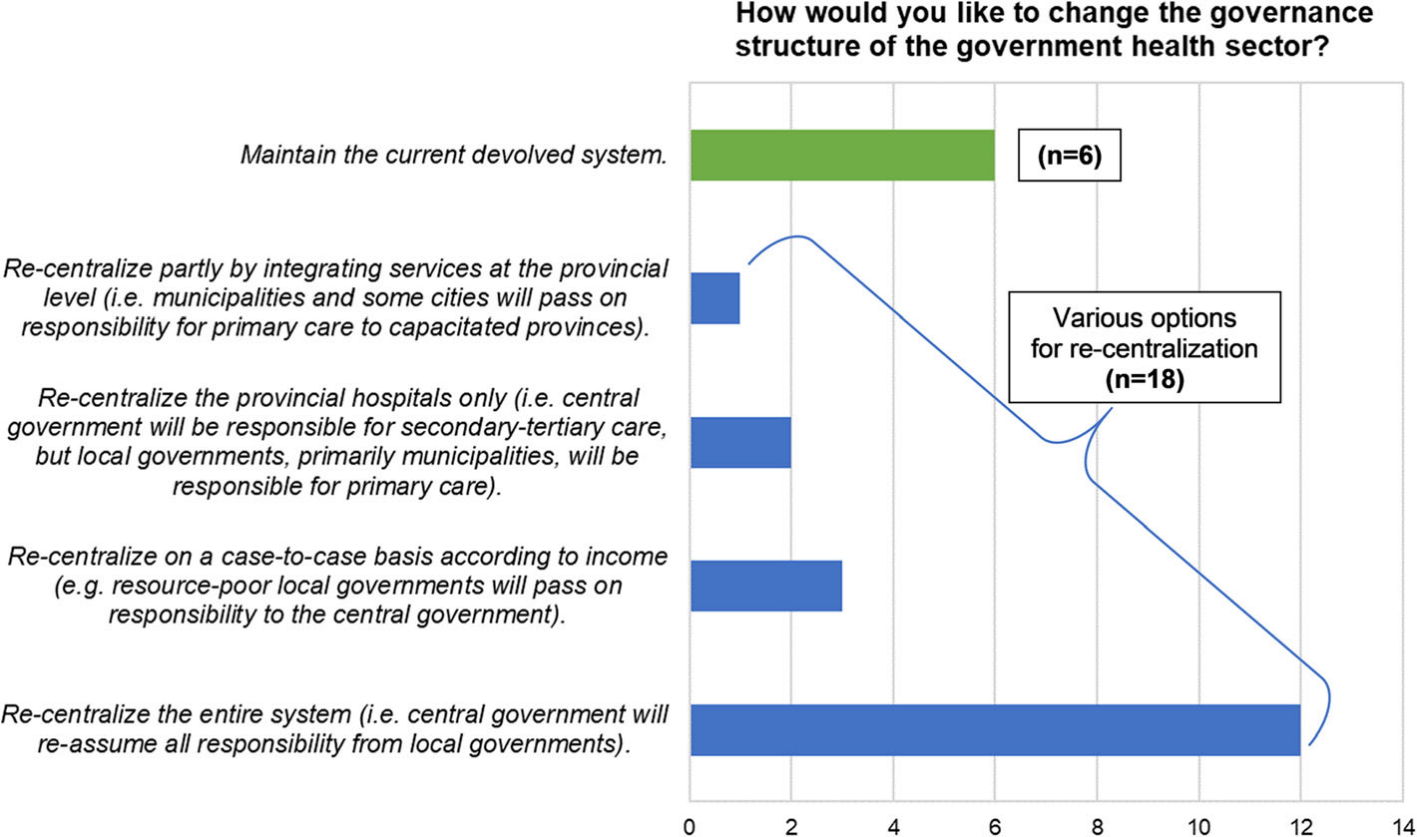
Survey participants who preferred maintaining the current devolved structure of governance and those who preferred various options for re-centralization

Responses to the question on the structure of governance revealed that most survey participants (18/24) would consider various options for re-centralization should they be able change the situation. At least half of survey participants (12/24) preferred the most extreme option of re-centralizing the entire system where the central government would recover ownership and management of local government health facilities and staff (Fig. 4).

Those who preferred maintaining devolution explained that *“the potential gains from devolution still outweigh the existing problems and inefficiencies,”* and that *“majority [of local governments] are supportive of the national health thrust especially if these are well promoted and explained by DOH representatives [i.e. DMOs].”* When asked what could improve the current devolved system, one responded with *“enlightening and empowering the local politicians about their respective health situations,”* and another answered that *“the DOH devolved the personnel, facilities, and equipment but not the budget for MOOE [maintenance and other operating expenses]”* and that the DOH should also transfer this budget to the local governments.

Conversely, the expressed preference to re-centralize government health services could be explained by the discontent among local health managers which stemmed from the inconsistency of benefits for local government human resources for health and the politicization in their management. Examples of human resources issues cited by survey respondents as their reasons for their desire to re-centralize include:

*“So that all the benefits will be given to the health personnel and their supervision will be under the DOH and not influenced by politics.” – Municipal health officer, 28 years in government.*

*“So that hiring of staff will be based on the qualifications and not on who they know in government.” – DOH public health associate, 1 year in government.*

*“Too much politicking in the local government. Health workers are being demoralized as benefits like hazard pay are not being given.” – Municipal health officer, 26 years in government.*

*“Some benefits are not approved due to reasons that the local government has no budget and the compensation for municipal staff is far lower compared to staff at the central level, yet local staff do have overloaded designations added to their work.” – DOH nurse deployed to the local government, 9 years in government.*

The other explanation provided by survey participants for their preference to re-centralize was what they perceived as overdependence on central support, as illustrated by the following answers:

*“The main source of medicines, vaccines, trainings and augmentation of human resources is the DOH, but the direction of local health services is controlled by the local politician. With full re-centralization of the health system… the resources from DOH will be maximized and used according to their purpose… There will be systematic and organized mobilization of resources.” – DOH development management officer, 22 years in government.*

*“Since local governments depend a lot on the DOH augmentation, I would rather prefer the entire system to be re-centralized so that all the needs of government health facilities will be provided, including the benefits and incentives of their human resources.” – DOH development management officer, 33 years in government.*

In contrast, the following quote from our interview with a former provincial governor may also offer the perspective of a political decision-maker:

*“[Re-centralization] will be a retrogression because it would run counter to the philosophy that decisions are best made at the local level as they are the ones down there who know the problem… So, empower the local governments. If the central government continues to be the source of programs and funding, we will not be able to develop the local communities and the local leaders… Health is a basic service that the local governments must provide, unless the local governments cannot be trusted to take care of it. But that’s a centralist point of view, and untrusting of the people’s potential to take care of themselves.” – Former provincial governor, 17 years in government.*

## Discussion

The objective of this study was to analyze decision-makers’ perspectives and preferences on who should be making decisions for the functions of health services at local levels and on their preferred structure of governance, and explored potential reasons for their preferences. While decentralization is a complex process that is often difficult to assess [32], unpacking it according to the functions that involved decision-making would be a useful step in analyzing it. Indeed, it would be inadequate to discuss decentralization or devolution only in broad terms. It would rather be meaningful to recognize that local level decision-making involves multiple actors in practice and figure out who among such actors would be in a better position to use the decision space granted by decentralization.

In other decentralized settings, discontent among human resources for health due to their lack of compensation and benefits in the wake of devolution has also been reported, such as in local governments’ failure to pay for the salaries of local health managers in Nigeria [33]. A review of case studies in several countries has also reported on how central employees got compensated differently from decentralized employees, and yet equitable remuneration is important for motivating health staff [34]. In decentralization, it is therefore paramount to consider health workers’ satisfaction as one of the potential indicators of effectiveness.

Dependence on central level support in decentralization has similarly been reported in Indonesia, an archipelago like the Philippines, where many functions have been transferred to local levels, but only those localities with revenues available from their own sources fared well in performing the functions [35]. Thus, like in the Philippines, the central government in Indonesia supports many local governments with grants, which increased their reliance on central support [36].

The preference to shift decision-making away from the local politician and more towards the local health officer was likely influenced by the expectation among survey respondents that the shift would rectify the perceived politicization in decision-making for the functions and the continued reliance on central support. The illustrative quotes from the interviews above showed examples of difficulties in upgrading facilities (resource management), hiring the qualified people for the job (human resources for health), and implementing initiatives for health promotion (health service delivery) when final decisions had to be made by the governor or mayor and not by the local health officer who had the technical capacity for these functions. The quotes further illustrated challenges in planning and financing when the local politician was either disinterested or not prioritizing health services. Hereby it was evident that decentralization could be challenging when political and technical decision-making were mixed. Supervision and management of local health services would certainly require technical knowhow and may be better left to the purview of those with training for and appreciation of public health services. Yet the way decentralization was carried out in the Philippines granted decision-making authority for most functions to the local politician, who may not always have the best interests of public health in mind.

Previous studies on devolution in the Philippines have already reported on the common tension between the local health officer and the local politician [37], or how the local politicians’ decisions were driven by their desire to get re-elected in office [38] yet continued to have substantial discretion in planning and budgeting for health services [39] and had a tendency to invest in tangible projects rather than support capacity building and system improvement [40]. Such observations in the Philippines are not unique. Other studies have likewise reported on how, for example, tribal affiliations rather than qualifications influenced the hiring of staff by local governments in Kenya [41], or how decisions for local health services in Indonesia and Kenya were made based on their appeal to the electorate [42, 43], or how political interference in health planning in Tanzania led to a complex relationship between the local politician and the local government technocrat [44].

Data management and monitoring, unlike the other functions, often did not involve the exercise of wielding power to increase electability in office, benefits which would often attract the interest of politicians. This may potentially explain why this study indicated that the current and desired decision-maker for this function was the same (i.e. the local health officer). A previous comprehensive study on decentralization of health information system in Tanzania, for example, did not report political interference in data management as a concern [45].

The preference for re-centralization in this study would appear inconsistent with the more common desire to expand decentralization even further in low- and middle-income countries (LMICs). In Kenya, a country with a much younger history of devolution since 2013, one study described how devolution ironically decreased local level decision space and recommended transferring more autonomy to county hospitals [46]. Other studies likewise reported a preference to expand decentralization by increasing capacities in Tanzania [47] and India [17], and by widening local decision space further for human resources management in Ghana [48] and Uganda [13], and for several functions in Fiji [16].

Why do our findings seem to contrast with the common desire for further decentralization in LMICs? First, it’s important to highlight that the perspectives expressed by study participants have drawn from a much longer history of devolution in the Philippines (~ 25 years). Second, given this long history, we postulate that local decision-makers may have somewhat lost their optimism that the devolved governance would still achieve the promise of health sector reform. This frustration with the system, driven by their perceived politicization of decision-making and the dependence of local governments on central augmentation may have led to their preference to re-centralize the system. It is, however, uncertain if re-centralization would solve the problems of politicization and overreliance on central support. If there is one thing to learn about decentralization, it is the realization that changing the structure of governance would not improve the health system unless certain conditions are met [18]. One such condition is strengthening both the supportive and regulatory roles of the central decision-maker (e.g. Ministry of Health) in assisting local governments to deliver quality health services, and in holding them accountable for meeting the targets set for the health system.

The phenomenon where devolution led to the concentration of power with politicians rather than truly bring decision-making closer to people has been called elite capture [49]. In Kenya, for example, the rapid transition to devolution led to a lack of clarity of roles and mistrust between health system actors, which allowed decision-making to be captured by local elites who favored political interests and populist priorities rather than genuine health needs [50]. A study in Pakistan suggested that, in order to manage political interference, the central ministry ought to deliver new roles in order to build consensus in the system and safeguard against local political pressures [51]. In relation to our findings, this would imply that the central level in the Philippines ought to continue supporting local governments but also take it upon itself the duty to promote and protect the rights and privileges of local human resources for health. Therefore, rather than re-centralize the Philippine health system—a change so radical that it would likely be politically-difficult to pursue—a policy option for consideration would be to maintain devolution but minimize politicization in decision-making. This could be achieved if the central level would further incentivize local governments’ satisfactory management of their human resources for health, and likewise further regulate decision-making for the various functions by including a positive assessment of performance in these functions as a condition in its licensing and accreditation of local government health facilities (i.e. a “carrot and stick” approach). In other words, it would be a decentralized or devolved system but with a revitalized central role for oversight and regulation, such that local politicians would still perform decision-making for the functions of local health services, but their decisions would be kept in check by a competent local health officer protected by the central government, as well as by the incentives and regulatory mechanisms imposed in the system by the central government to ensure good decision-making across the functions.

### Limitations

Albeit several reminders were sent, the response rate from the online survey was unsatisfactorily low. This is a limitation of the study which also suggests that we do not have the full picture of decision-makers’ perspectives in the Philippines. Possible reasons for the low response rate may include limited access to the internet in certain areas, the lack of time for potential respondents to consult and respond to their emails given their heavy workload, or simply the lack of interest from certain decision-makers to participate. Local politicians were under-represented despite this study having been endorsed by the DOH regional office. Further studies may therefore consider implementing the survey via phone calls, or even face-to-face, as this might increase the response rate, although such will require more time and resources, while accessing politicians and getting them to participate is often easier said than done. Expanding from this study, it would be interesting to see if subsequent and larger surveys would reveal a different pattern of preferences should more politicians participate as they, we hypothesize, might likely express a desire to maintain devolution.

## Conclusion

Decentralization is a process which several countries have chosen to undertake. The fact that decision-making authority now belongs to local levels after decentralization or devolution may be a desired outcome in itself. However, as suggested by our study in the Philippines, this transfer does not necessarily ensure that effective decisions for local health services are made. Rather than re-centralize, a policy option for the Philippines includes emphasizing the role of the central government in exercising its regulatory oversight over local governments to minimize political interference in decision-making and to protect the welfare of local government technical staff.

Decentralization or devolution of health services, whether in the Philippines or elsewhere, must not only be satisfied with the outcome that local levels have been granted the space to make decisions for themselves. Given the many actors at local levels, who should decide for health services? Pursue decentralization indeed—but it’s critical to ensure that those who were granted with the decision space actually make decisions for the various functions with adequate capacities and are able to grasp the importance of health services.

## Supplementary information

**Additional file 1.** Online survey questionnaire

**Additional file 2.** Anonymized dataset of responses to the online survey

## Data Availability

All relevant data are provided in the manuscript, in the online supplementary files (Additional files 1 and 2), and in two other publications which have been referenced [18, 24].

## Abbreviations

DMO: Development Management Officer
DOH: Department of Health
DTTB: Doctors to the Barrios
HFEP: Health Facilities Enhancement Program
LHB: Local Health Board
LMICs: Low- and Middle-Income Countries
MOOE: Maintenance and Other Operating Expenses
NDP: Nurse Deployment Program
PHA: Public Health Associate
PhilHealth: Philippine Health Insurance Corporation
RHU: Rural Health Unit
TB-DOTS: Tuberculosis, Directly-Observed Treatment, Short-Course
USD: United States Dollar
