# Supplementary material for: Who should decide for local health services? A mixed methods study of preferences for decision-making in the decentralized Philippine health system
Source: BMC Health Serv Res. 2020 Apr 15;20:305. doi: 10.1186/s12913-020-05174-w (PMC7158124; doi:10.1186/s12913-020-05174-w)
Supplement: Supplementary file 1 — Additional file 1. Online survey questionnaire [file 12913_2020_5174_MOESM1_ESM.pdf]

Survey of perspectives on health sector devolution

You have received this email because you are being invited to participate in an online survey on the perspectives of local decision-makers on health sector devolution. This survey will take only 10 minutes.

This research is being led by Dr. Harvy Joy Liwanag and Prof. Kaspar Wyss of the Swiss Tropical and Public Health Institute.

Please click continue/next (or click the link above) for more information.

* Required

Informed consent

In 1992, the Local Government Code was implemented in the Philippines which transferred decision-making authority over local health services from the national government to the local government units. We are interested to know about how devolution has affected the health sector at local levels.

You are being invited to take part in this research because we feel that your experience as a decision-maker in the health sector can contribute to our analysis of devolution in the Philippines.

Your participation in this research is entirely voluntary. If you accept our invitation, you will answer an online survey that will take 10 minutes.

The information that we will collect from this survey will be strictly confidential. Your name will not be recorded and your answers will be stored securely and accessible only to the Principal Investigator.

There will be no direct benefit to you if you answer this survey, but your participation is likely to help us find out how to improve the implementation of devolution in the Philippine health sector.

To continue, please click next.

Ethics approval

This research has been reviewed and approved by the ethics review committee of Northwest and North Central Switzerland (EKNZ) and the National Ethics Committee (NEC) of the Philippines (NEC code: 2016-013). If you wish to know more about the NEC, you may contact:

National Ethics Committee
Philippine Council for Health Research and Development
Bicutan, Taguig City, Metro Manila
Tel. nos.: (02) 837 7537 or (02) 837 2071 to 82 local 2112
Email address: nationalethicscommittee.ph@gmail.com

If you have any questions, you may reach the Principal Investigator through the following contact information:

Dr. Harvy Joy Liwanag
Swiss Tropical and Public Health Institute, Basel, Switzerland
and
Ateneo School of Medicine and Public Health, Pasig City, Philippines
Email addresses: harvy.liwanag@swisstph.ch or harvyliwanagmd@gmail.com

By continuing to the next page, you confirm that you have understood the information provided and agree to participate in this survey.

To answer the survey, please click next.

Participant's profile

1. **What is your present role in the health sector of Region 1? Choose one. (If retired/resigned/transferred staff, please indicate the last position in Region 1.)** *
Mark only one oval.
   - Development Management Officer (DMO) or "DOH Rep"
   - Provincial Health Officer (PHO)
   - Municipal Health Officer (MHO)
   - City Health Officer (CHO)
   - Chief of Hospital (e.g. Provincial/District/City Hospital, etc.)
   - "Doctor to the Barrio" (DTTB)
   - Provincial Governor
   - Municipal Mayor
   - City Mayor
   - Other: ______

2. **What is your main profession?** *
Mark only one oval.
   - Medical doctor
   - Nurse
   - Midwife
   - Pharmacist
   - Medical technologist
   - Dentist
   - Other: ______

3. **Please indicate your sex.** *
Mark only one oval.
   - Female
   - Male

4. **How old are you (in years)?** *

______

5. **How many years have you been serving in the government/public health sector?** *

______

6. **At present, which province are you serving? (For retired/resigned/transferred staff, choose previous province of assignment.)** *
Mark only one oval.
   - Ilocos Norte
   - Ilocos Sur
   - La Union
   - Pangasinan
   - All of these 4 provinces

ONLY for those serving in ILOCOS NORTE:

If not applicable, skip to next page.

7. **Which municipality/city are you MAINLY serving? Check all that apply. (For retired/resigned/transferred staff, choose last place of assignment.)**
Check all that apply.

- [ ] Adams
- [ ] Bacarra
- [ ] Badoc
- [ ] Bangui
- [ ] Banna
- [ ] Batac City
- [ ] Burgos
- [ ] Carasi
- [ ] Currimao
- [ ] Dingras
- [ ] Dumalneg
- [ ] Laoag City
- [ ] Marcos
- [ ] Nueva Era
- [ ] Pagudpud
- [ ] Paoay
- [ ] Pasuquin
- [ ] Piddig
- [ ] Pinili
- [ ] San Nicolas
- [ ] Sarrat
- [ ] Solsona
- [ ] Vintar
- [ ] ALL OF THE ABOVE
- [ ] Other: ____________________

ONLY for those serving in ILOCOS SUR:
If not applicable, skip to next page.

8. **Which municipality/city are you MAINLY serving? Check all that apply. (For retired/resigned/transferred staff, choose last place of assignment.)**
Check all that apply.

- [ ] Alilem
- [ ] Banayoyo
- [ ] Bantay
- [ ] Burgos
- [ ] Cabugao
- [ ] Candon City
- [ ] Caoayan
- [ ] Cervantes
- [ ] Galimuyod
- [ ] Gregorio del Pilar
- [ ] Lidlidda
- [ ] Magsingal
- [ ] Nagbukel
- [ ] Narvacan
- [ ] Quirino
- [ ] Salcedo
- [ ] San Emilio
- [ ] San Esteban
- [ ] San Ildefonso
- [ ] San Juan
- [ ] San Vicente
- [ ] Santa
- [ ] Santa Catalina
- [ ] Santa Cruz
- [ ] Santa Lucia
- [ ] Santa Maria
- [ ] Santiago
- [ ] Santo Domingo
- [ ] Sigay
- [ ] Sinait
- [ ] Sugpon
- [ ] Suyo
- [ ] Tagudin
- [ ] Vigan City
- [ ] ALL OF THE ABOVE
- [ ] Other: ____________________

ONLY for those serving in LA UNION:

If not applicable, skip to next page.

9. **Which municipality/city are you MAINLY serving? Check all that apply. (For retired/resigned/transferred staff, choose last place of assignment.)**
Check all that apply.

- [ ] Agoo
- [ ] Aringay
- [ ] Bacnotan
- [ ] Bagulin
- [ ] Balaoan
- [ ] Bangar
- [ ] Bauang
- [ ] Burgos
- [ ] Caba
- [ ] Luna
- [ ] Naguilian
- [ ] Pugo
- [ ] Rosario
- [ ] San Fernando City
- [ ] San Gabriel
- [ ] San Juan
- [ ] Santo Tomas
- [ ] Santol
- [ ] Sudipen
- [ ] Tubao
- [ ] ALL OF THE ABOVE
- [ ] Other: ____________________

ONLY for those serving in PANGASINAN:

If not applicable, skip to next page.

10. **Which municipality/city are you MAINLY serving? Check all that apply. (For retired/resigned/transferred staff, choose last place of assignment.)**

Check all that apply.

- [ ] Agno
- [ ] Aguilar
- [ ] Alaminos City
- [ ] Alcala
- [ ] Anda
- [ ] Asingan
- [ ] Balungao
- [ ] Bani
- [ ] Basista
- [ ] Bautista
- [ ] Bayambang
- [ ] Binalonan
- [ ] Binmaley
- [ ] Bolinao
- [ ] Bugallon
- [ ] Burgos
- [ ] Calasiao
- [ ] Dagupan City
- [ ] Dasol
- [ ] Infanta
- [ ] Labrador
- [ ] Laoac
- [ ] Lingayen
- [ ] Mabini
- [ ] Malasiqui
- [ ] Manaoag
- [ ] Mangaldan
- [ ] Mangatarem
- [ ] Mapandan
- [ ] Natividad
- [ ] Pozorrubio
- [ ] Rosales
- [ ] San Carlos City
- [ ] San Fabian
- [ ] San Jacinto
- [ ] San Manuel
- [ ] San Nicolas
- [ ] San Quintin
- [ ] Santa Barbara
- [ ] Santa Maria
- [ ] Santo Tomas
- [ ] Sison
- [ ] Sual

- [ ] Tayug
- [ ] Umingan
- [ ] Urbiztondo
- [ ] Urdaneta City
- [ ] Villasis
- [ ] ALL OF THE ABOVE
- [ ] Other: ______________________

Understanding of devolution

11. **In one sentence, could you please explain what devolution means for you? (Devolution is. . .)** *

Benefits of devolution

12. **The perceived benefits of devolution to the health sector are many. Drawing from your experience, which among the following is the MOST IMPORTANT benefit?** *
Mark only one oval.

- ( ) Empowerment of local governments to decide for themselves and address their own health needs
- ( ) Increased participation of communities in decision-making for health
- ( ) Development of local health programs that match local health needs or are appropriate to the local context
- ( ) Faster health service delivery at local levels without the need to wait for decisions from the national government
- ( ) More efficient administration or operation of local health services
- ( ) No benefit to the health sector
- ( ) Other: ______________________

Challenges in devolution

13. **The perceived challenges in implementing devolution are also many. Drawing from your experience, which among the following is the MOST DIFFICULT challenge in implementing devolution?** *

Mark only one oval.

- Decisions related to local health services have become politicized instead of evidence-based.
- Local health facilities have deteriorated in quality.
- Local health workers' full range of compensation and benefits have not been provided consistently.
- Many areas lack the number of health workers required to meet the needs of the local population.
- Complete and timely collection of health-related data has been difficult to achieve.
- Local governments have continued to depend on assistance from the DOH.
- Local health services have not been prioritized in the local government budget.
- No challenge in implementing devolution.
- Other: ______________________

Planning

14. **Drawing from your experience, who has the MOST influence in developing local health plans (e.g. plans discussed in the Local Health Board or those that become the "Investment Plan for Health")?** *

Mark only one oval.

- Local chief executive (i.e. governor or mayor)
- Local health officer (PHO, MHO, or CHO)
- DMO or "DOH Rep"
- DOH Regional Director
- Representative of Zuellig Family Foundation
- Provincial/Municipal/City Budget Officer
- Provincial Board Member or Municipal/City Councilor
- PhilHealth representative
- Other: ______________________

15. **If you could change the situation, WHO SHOULD control the development of local health plans?** *

Mark only one oval.

- Local chief executive (i.e. governor or mayor)
- Local health officer (PHO, MHO, or CHO)
- DMO or "DOH Rep"
- DOH Regional Director
- Representative of Zuellig Family Foundation
- Provincial/Municipal/City Budget Officer
- Provincial Board Member or Municipal/City Councilor
- PhilHealth representative
- Other: ______________________

Health financing

16. **Drawing from your experience, who has the MOST influence in determining the local health budget, or how to spend the funds for local health services?** *

Mark only one oval.

- Local chief executive (i.e. governor or mayor)
- Local health officer (PHO, MHO, or CHO)
- DMO or "DOH Rep"
- DOH Regional Director
- Representative of Zuellig Family Foundation
- Provincial/Municipal/City Budget Officer
- Provincial Board Member or Municipal/City Councilor
- PhilHealth representative
- Other: ____________________

17. **If you could change the situation, WHO SHOULD control the local health budget, or how to spend the funds for local health services?** *

Mark only one oval.

- Local chief executive (i.e. governor or mayor)
- Local health officer (PHO, MHO, or CHO)
- DMO or "DOH Rep"
- DOH Regional Director
- Representative of Zuellig Family Foundation
- Provincial/Municipal/City Budget Officer
- Provincial Board Member or Municipal/City Councilor
- PhilHealth representative
- Other: ____________________

Resource management

18. **Drawing from your experience, who has the MOST influence in making decisions in the maintenance/upgrade of local health facilities, as well as in purchasing equipment and supplies (e.g. medicines)?** *

Mark only one oval.

- Local chief executive (i.e. governor or mayor)
- Local health officer (PHO, MHO, or CHO)
- DMO or "DOH Rep"
- DOH Regional Director
- Representative of Zuellig Family Foundation
- Provincial/Municipal/City Budget Officer
- Provincial Board Member or Municipal/City Councilor
- PhilHealth representative
- Other: ____________________

19. **If you could change the situation, WHO SHOULD control the decisions in the maintenance/upgrade of local health facilities, as well as in purchasing of equipment and supplies (e.g. medicines)?** *

Mark only one oval.

- Local chief executive (i.e. governor or mayor)
- Local health officer (PHO, MHO, or CHO)
- DMO or "DOH Rep"
- DOH Regional Director
- Representative of Zuellig Family Foundation
- Provincial/Municipal/City Budget Officer
- Provincial Board Member or Municipal/City Councilor
- PhilHealth representative
- Other: ______________________

Human resources for health

20. **Drawing from your experience, who has the MOST influence in the hiring and training of local health staff, and in deciding on what types of benefits these staff receive?** *

Mark only one oval.

- Local chief executive (i.e. governor or mayor)
- Local health officer (PHO, MHO, or CHO)
- DMO or "DOH Rep"
- DOH Regional Director
- Representative of Zuellig Family Foundation
- Provincial/Municipal/City Budget Officer
- Provincial Board Member or Municipal/City Councilor
- PhilHealth representative
- Other: ______________________

21. **If you could change the situation, WHO SHOULD control the hiring and training of local health staff, and in deciding on what types of benefits these staff receive?** *

Mark only one oval.

- Local chief executive (i.e. governor or mayor)
- Local health officer (PHO, MHO, or CHO)
- DMO or "DOH Rep"
- DOH Regional Director
- Representative of Zuellig Family Foundation
- Provincial/Municipal/City Budget Officer
- Provincial Board Member or Municipal/City Councilor
- PhilHealth representative
- Other: ______________________

Health service delivery

22. **Drawing from your experience, who has the MOST influence in deciding which health programs/projects (either DOH programs or LGU-initiated programs) will be implemented in the locality?** *

Mark only one oval.

- Local chief executive (i.e. governor or mayor)
- Local health officer (PHO, MHO, or CHO)
- DMO or "DOH Rep"
- DOH Regional Director
- Representative of Zuellig Family Foundation
- Provincial/Municipal/City Budget Officer
- Provincial Board Member or Municipal/City Councilor
- PhilHealth representative
- Other: ______________________

23. **If you could change the situation, WHO SHOULD decide which health programs/projects (either DOH programs or LGU-initiated programs) will be implemented in the locality?** *

Mark only one oval.

- Local chief executive (i.e. governor or mayor)
- Local health officer (PHO, MHO, or CHO)
- DMO or "DOH Rep"
- DOH Regional Director
- Representative of Zuellig Family Foundation
- Provincial/Municipal/City Budget Officer
- Provincial Board Member or Municipal/City Councilor
- PhilHealth representative
- Other: ______________________

Data management and monitoring

24. **Drawing from your experience, who has the MOST influence in deciding which data to collect and how to assess if the local government is performing well in terms of health?** *

Mark only one oval.

- Local chief executive (i.e. governor or mayor)
- Local health officer (PHO, MHO, or CHO)
- DMO or "DOH Rep"
- DOH Regional Director
- Representative of Zuellig Family Foundation
- Provincial/Municipal/City Budget Officer
- Provincial Board Member or Municipal/City Councilor
- PhilHealth representative
- Other: ______________________

25. **If you could change the situation, WHO SHOULD decide which data to collect and how to assess if the local government is performing well in terms of health?** *

Mark only one oval.

- Local chief executive (i.e. governor or mayor)
- Local health officer (PHO, MHO, or CHO)
- DMO or "DOH Rep"
- DOH Regional Director
- Representative of Zuellig Family Foundation
- Provincial/Municipal/City Budget Officer
- Provincial Board Member or Municipal/City Councilor
- PhilHealth representative
- Other: ____________

Future directions

26. **Reflecting on your experience in devolution, what would like to happen with the local health system?** *

Mark only one oval.

- Maintain the current devolved system. *Skip to question 27.*
- Re-centralize the entire health system (i.e. DOH will re-absorb everything from provincial/district hospitals to RHUs, including staff). *Skip to question 29.*
- Re-centralize the health system, but up to the provincial level only (i.e. DOH will absorb the provincial health office and provincial/district hospitals but the local governments will maintain the RHUs). *Skip to question 30.*
- Devolve up to the provincial level only (i.e. the provincial government will maintain the provincial/district hospitals and will also take over the RHUs from the municipalities/cities). *Skip to question 31.*
- Re-centralize on a case-to-case basis according to income (i.e. local health facilities in wealthy local governments will remain devolved while those from poor local governments will be absorbed by the DOH). *Skip to question 32.*
- I have a different suggestion. *Skip to question 33.*
- Undecided *Skip to question 34.*

Maintain the current devolved system

27. **Please explain (in one sentence) why you wish to maintain the current devolved system.** *

____

28. **If your desire is to maintain the current devolved system, please suggest one very important step to improve local health service delivery given the current system.** *

____

Skip to "Conclusion."

Re-centralize the entire health system

i.e. DOH will re-absorb everything from RHUs to provincial/district hospitals, including staff

29. **Please explain (in one sentence) why you wish to re-centralize the entire health system? ***

Skip to "Conclusion."

Re-centralize the health system, but up to the provincial level only

i.e. DOH will absorb the provincial health office and provincial/district hospitals but the local governments will maintain the RHUs

30. **Please explain (in one sentence) why you wish to re-centralize the health system up to the provincial level only. ***

Skip to "Conclusion."

Devolve up to the provincial level only

i.e. the provincial government will maintain the provincial/district hospitals and will also take over the RHUs from the municipalities/cities

31. **Please explain (in one sentence) why you wish to devolve up to the provincial level only. ***

Skip to "Conclusion."

Re-centralize on a case-to-case basis according to income

i.e. local health facilities in wealthy local governments will remain devolved while those from poor local governments will be absorbed by the DOH

32. **Please explain (in one sentence) why you wish to re-centralize on a case-to-case basis according to income. ***

Skip to "Conclusion."

I have a different suggestion

33. **Please explain (in one sentence) your suggestion.** *

Skip to "Conclusion."

Undecided

34. **Please explain (in one sentence) why you are undecided.** *

Skip to "Conclusion."

Conclusion

This is the end of the survey, and we greatly appreciate your time. Thank you very much!

Results will be shared through Dr. Myrna Cabotaje and the DOH Regional Office 1 as soon as these are available.

If you are ready to finalize and submit your answers, please click submit.

Powered by
Google Forms
